# The proteomic landscape of microglia in health and disease

**DOI:** 10.3389/fncel.2024.1379717

**Published:** 2024-03-15

**Authors:** Emma Davis, Amy F. Lloyd

**Affiliations:** ^1^The Francis Crick Institute, London, United Kingdom; ^2^UK Dementia Research Institute at UCL, University College London, London, United Kingdom; ^3^Cell Signalling and Immunology, University of Dundee, Dundee, United Kingdom

**Keywords:** microglia, neuroscience, neurodegeneration, mass spectrometry, proteomics

## Abstract

Microglia are the resident immune cells of the central nervous system (CNS) and as such play crucial roles in regulating brain homeostasis. Their presence in neurodegenerative diseases is known, with neurodegeneration-associated risk genes heavily expressed in microglia, highlighting their importance in contributing to disease pathogenesis. Transcriptomics studies have uncovered the heterogeneous landscape of microglia in health and disease, identifying important disease-associated signatures such as DAM, and insight into both the regional and temporal diversity of microglia phenotypes. Quantitative mass spectrometry methods are ever increasing in the field of neurodegeneration, utilised as ways to identify disease biomarkers and to gain deeper understanding of disease pathology. Proteins are the main mechanistic indicators of cellular function, yet discordance between transcript and proteomic findings has highlighted the need for in-depth proteomic phenotypic and functional analysis to fully understand disease kinetics at the cellular and molecular level. This review details the current progress of using proteomics to define microglia biology, the relationship between gene and protein expression in microglia, and the future of proteomics and emerging methods aiming to resolve heterogeneous cell landscapes.

## Introduction

Microglia are specialised cells that act as the primary form of innate immune defence in the brain and spinal cord. Originating from the yolk sac during early embryonic development (Ginhoux et al., 2010), microglia play critical roles in both CNS development and maintenance of brain homeostasis. They are also key players in neuroprotection; slice culture live imaging has captured their highly motile processes extending and retracting to show their constant surveying of their local environment (Lloyd et al., 2019), enabling them to rapidly assess and respond to injury or disease. Upon detecting damage or pathogens, microglia are able to rapidly respond by downregulating their homeostatic markers associated with surveillance, including P2RY12, TMEM119 and CX3CR1 (Zrzavy et al., 2017; Butovsky and Weiner, 2018), and adopt a plethora of stimulus-dependant phenotypes and functions. For example, in response to demyelination, microglia phagocytose myelin debris and increase expression of pro-inflammatory inducible nitric oxide synthase (iNOS) and tumour necrosis factor alpha (TNFα). These cytokines represent essential ‘alarms’ for neighbouring microglia to further promote debris clean-up, as well as promote the recruitment of oligodendrocyte precursor cells (OPC) to the area to initiate remyelination (Miron et al., 2013; Lloyd et al., 2019). Microglia inflammation is then attenuated and replaced by insulin-like growth factor 1 (IGF-1) expressing microglia to promote remyelination (Miron et al., 2013; Lloyd et al., 2019). These findings exemplify the diverse phenotypes and functions of microglia, and the importance of tight regulation of these responses. Indeed, the consequences of dysregulated microglia responses is evidenced by failed remyelination in mouse models and tissue from multiple sclerosis (MS) patients (Miron et al., 2013; Lloyd et al., 2019). Furthermore, dysregulated microglia seen as a prominent pathological feature of other neurodegenerative diseases, such as Alzheimer’s Disease (AD). Understanding how microglia become dysregulated and how to prevent microglia from becoming chronically inflamed is of great importance for novel therapeutic development.

Our understanding of microglia phenotypes and responses in neurodegenerative diseases in recent years has been significantly derived from transcriptomic studies. Indeed, single cell transcriptomics has uncovered the heterogenous landscape of microglia both in health and disease (Hammond et al., 2019; Masuda et al., 2019; Olah et al., 2020; Mancuso et al., 2022). However, to understand the nuances of signalling and post-translational modifications, particularly for identifying druggable targets, it is essential that we understand these cells at their protein level. As the field now turns its attention to utilising mass spectrometry to analyse microglia, this review discusses how proteomics has furthered our understanding of microglia biology and signalling both in homeostasis and in the context of neurodegenerative diseases.

### Proteomics: advantages and limitations

The study of protein expression in cells is not a new concept, however the more unbiased approach of uncovering the whole proteome using mass spectrometry has enabled us to gain a deeper understanding of cell signalling, metabolomics and functional responses, and has gained significant traction over the last decade. This has been outshined however by the popularity of transcriptomics which has showcased the complexity and diversity of microglia phenotypes throughout the brain in health and disease. Single cell RNA sequencing (scRNAseq) has highlighted both the regional diversity of microglia in the brain, and the heterogenous responses in disease. Characterisation of disease-associated microglia (DAM) (Keren-Shaul et al., 2017), highlights the breakthroughs achieved by transcriptomics in our understanding of microglia plasticity and their responses in disease.

Despite the importance of these landmark studies, it is evident that mRNA expression is a poor correlate of protein expression (Bauernfeind and Babbitt, 2017; Johnson et al., 2022), therefore making it difficult to interpret cell phenotypes and function. This is particularly evident in AD studies, where comparisons between mRNA and protein networks from AD patients revealed significant correlative disparity (Johnson et al., 2022). This included a MAPK/metabolism protein module, the most highly correlated module to cognitive function in AD patients, that was not found at the transcript level (Johnson et al., 2022). These findings highlight the importance of proteomic analysis for understanding and predicting AD pathology and disease progression.

Proteomics offers several advantages over transcriptomics, including the ability to study post-translational modifications (PTMs), which significantly influence protein function and cannot be appreciated by transcriptomic analysis. These modifications are crucial in processes such as cell signalling, protein transport and enzyme activity, all of which play vital roles in immune modulation and inflammation (Hegde et al., 2003). Unlike transcriptomics, which can often be a delayed representation of cell behaviour, proteomics reflects the real-time status of cells or tissues, providing a more immediate snapshot of physiology or pathology.

There are some limitations to proteomic studies that must also be considered. Contamination from culture components such as foetal bovine serum (FBS), haemoglobin from red blood cell contamination, and keratin from sample handling can mask sample peptide detection. Isolation methods can also lead to differences in protein detection. For example, fluorescence-activated (FACS) and magnetic-activated cell sorting (MACS) of microglia populations prior to proteomic evaluation (Rayaprolu et al., 2020) lead to differential enrichment of protein groups. As MACS-sorted microglia proteomes show enrichment of non-microglia associated proteins, such as synaptic and myelin proteins, it is thought this difference is due to greater cellular contamination by MACS. Deconvolution of raw data is complex, with protein identification and quantitation variable depending on software used (including version differences of the same software), reference database used and completeness of species proteome annotation. Finally, despite methodological and technological progress, proteome resolution at the single cell level is significantly lagging behind transcriptomics. Proteomics usually represents an average across bulk grouping of cells, despite their known heterogeneity. It is therefore important to consider these challenges when designing experiments for proteomic analysis.

### Proteomic analysis of microglia identity

Microglia may represent a novel therapeutic target for many neurodegenerative diseases by modulation of their dysregulated inflammatory responses. To uncover druggable targets, it is essential to understand microglia protein expression profiles. Proteomic profiling of human myeloid cells derived from blood, CSF and brain using mass cytometry by time of flight (CyTOF) helped to uncover key protein markers to distinguish brain resident microglia from peripheral myeloid cells, as well as insight into microglia heterogeneity across brain regions (Bottcher et al., 2019). This included differential expression of protein markers P2RY12, TMEM119, EMR1, CD64 and TREM2 in microglia, and enrichment of CD44, CCR2, CD45, CD14 and CD16 in blood and CSF derived myeloid cells. Furthermore, distinct protein expression profiles were observed in microglia from different brain regions; microglia from the sub ventricular zone and thalamus expressed enrichment of proteins associated with microglia activation, including CD68, CD86, CD45 and HLA-DR (Bottcher et al., 2019). Although CyTOF is limited to selected markers of interest, our own lab has recently generated detailed proteomic maps of mouse and human microglia from *in vitro* and *ex vivo* sources using mass spectrometry (Lloyd et al., 2022). Proteomic evaluation of these microglia groups identified striking differences in protein expression between *in vitro* and *ex vivo* microglia from the same species. In both mouse and human, *in vitro* culture of microglia induced rapid protein mass increases along with increases in glycolysis, reduction of homeostatic markers and increased expression of DAM associated markers. Interestingly, these *in vitro*-induced proteomic changes were reversed once cells were back in the *in vivo* environment, as shown by xenograft microglia proteomes (Lloyd et al., 2022). These findings have been vital for our understanding of basal microglia proteomes and the suitability of model systems, as well as highlighting the plasticity of microglia proteomic reprogramming.

Proteomic analysis of male and female microglia has highlighted important sex-specific differences in translation. Approximately 10% of all proteins detected were differentially expressed between unstimulated male and female adult mouse microglia (Guneykaya et al., 2018). Most of these proteins were enriched in male microglia, including myosin proteins, purinergic receptors (including P2RY12), and toll-like receptors (TLRs), with female microglia having enrichment in type 1 interferon signalling. Proteomics has therefore provided important phenotypic and functional insight into microglia biology.

### Proteomic analysis of microglia in disease

**Figure 1:**
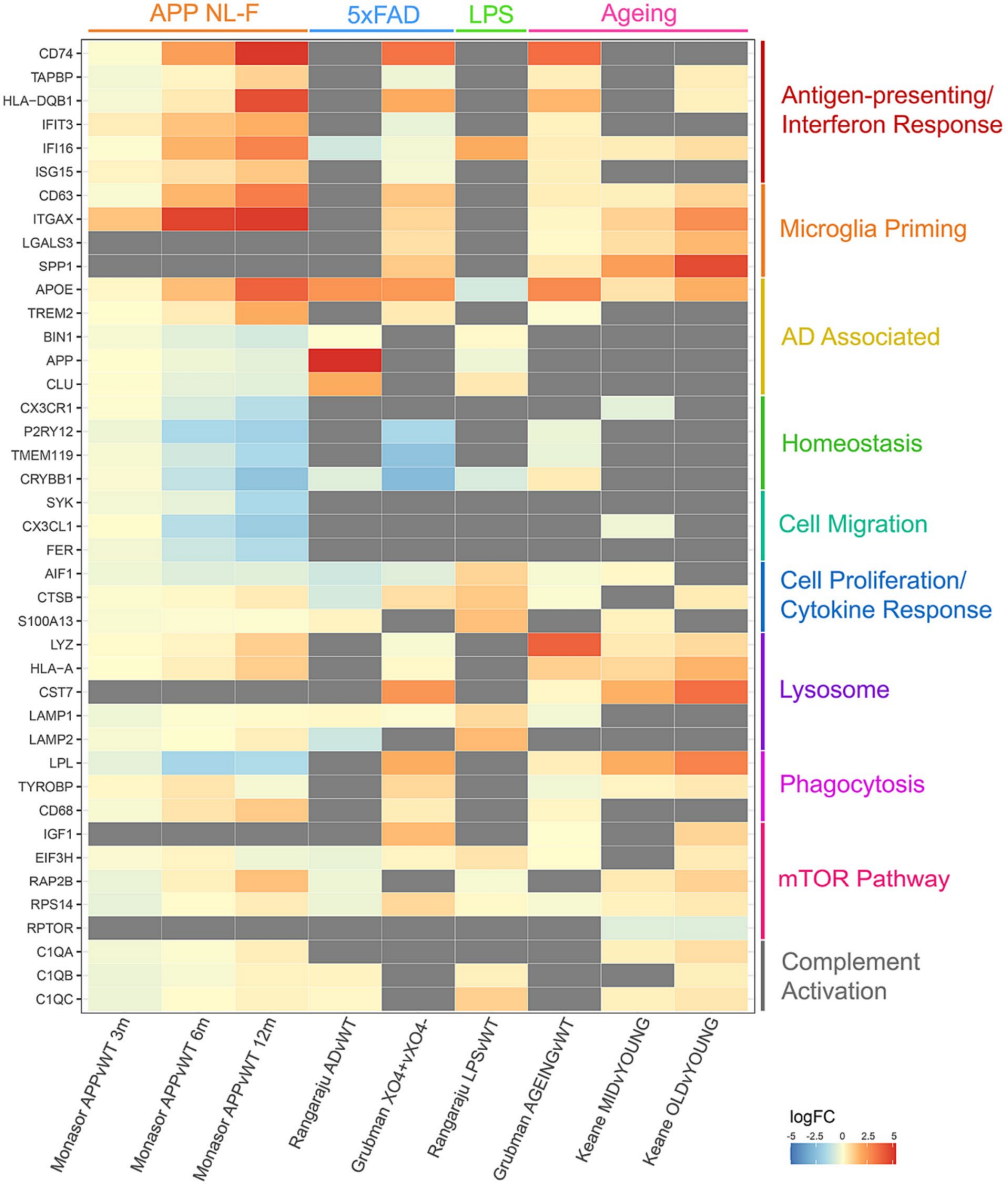
Meta-analysis of 4 different quantitative proteomics microglia mouse studies, comparing individual protein log fold changes across experimental conditions. Log fold changes were calculated by the studies referenced and merged on mouse-human orthologous proteins. Gray boxes indicate proteins that are not detected in individual studies.

The majority of microglia proteomic analysis has focused on protein changes induced by disease models or inflammatory stimuli. To analyse commonalities and differences, we have produced a meta-analysis of microglia from four quantitative mouse proteomics studies (Dan et al., 2008; Monasor et al., 2020; Grubman et al., 2021; Keane et al., 2021) (Figure 1). These studies include microglia from models of Alzheimer’s Disease (APP NL-F and 5xFAD), LPS-induced inflammation and ageing. Proteins up-regulated in microglia in disease compared to control are noted in red, whereas proteins down-regulated are shown in blue. The top most dysregulated proteins across studies were selected for the final meta-analysis and grouped by biological function to show how the main biological pathways in microglia are perturbed across different disease and stimuli contexts. The aim of this meta-analysis is to highlight the multifaceted response of the mouse microglia proteome consolidated from the few proteomics datasets currently available in this field that may pave the way for further interrogation and understanding of the human microglia proteome.

Comparisons of these proteomic datasets identify both context-specific and non-specific proteomic reprogramming in microglia. In all cases, where detected, microglia decrease expression of homeostatic markers, highlighting their ‘activated’ status regardless of context. Interestingly, increases in APOE expression are seen in ageing and AD models, but decreased expression is observed in response to LPS. Upregulation of antigen-presenting and interferon response proteins, and downregulation of proteins involved in microglial homeostasis and cell migration is seen in the APP mouse model of amyloid plaque development (Monasor et al., 2020). Plaque-associated microglia display a strong phagocytic phenotype with an upregulation of proteins involved in priming, whereas LPS treated microglia display a strong cell proliferation and cytokine response to neuroinflammation (Grubman et al., 2021). Dysregulated mTOR-signalling is observed with age, increasing translation of inflammatory mediators and thus priming microglia (Dan et al., 2008; Keane et al., 2021). Upregulation of antigen-presenting and interferon response proteins is observed in both studies comparing ageing against young microglia, as well as dysregulation in lysosomal proteins (Grubman et al., 2021; Keane et al., 2021). These datasets demonstrate the nuances in proteomic reprogramming of microglia in different contexts, demonstrating that such changes can be both conserved to microglia transitioning from a ‘homeostatic’ to ‘activated’ state and context specific.

#### Microglia in AD

GWAS studies suggest microglia play a crucial role in AD pathogenesis, with many AD risk genes being highly or selectively expressed by microglia (Kunkle et al., 2019; Wang et al., 2021; Kosoy et al., 2022). Increasing evidence suggests microglia play a key role in protecting against AD, as dysregulated microglial response to β-amyloid pathology is associated with increased AD risk (Davies et al., 2017). The role of microglia in AD is complex and can be seen as both beneficial and detrimental depending on context and disease stage. Microglia mediate synapse loss via engulfment of synapses, however dysregulated synaptic pruning by microglia can contribute to synapse loss and cognitive decline in AD (Hong et al., 2016; Hansen et al., 2017; Tzioras et al., 2023). Additionally, while microglia typically produce neurotrophic factors supporting neuron survival and repair, in AD this support may be compromised (Wang et al., 2023). It is therefore vital that we further understand the temporal phenotypes and responses of microglia throughout AD.

Microglia reacting to AD pathology can exhibit protective behaviour in the early stages of disease by phagocytosing amyloid-beta plaques and cellular debris, however these microglia can also contribute to oxidative stress by producing Reactive Oxygen Species (ROS), damaging neurons and other brain cells. XO4+ plaque-associated microglia isolated from 5xFAD 6 month old mice and processed for targeted liquid-chromatography sequential window acquisition of all theoretical spectra mass spectrometry (LC-SWATH-MS), revealed a loss of proteins involved in homeostasis and an upregulation of proteins involved in response to interferon-gamma, phagocytosis and regulation of cell migration (Grubman et al., 2021). Proteins down-regulated on both the transcript and protein level in XO4+ microglia include DOCK10, CRYBB1, PLXNA1 and FGD2, while lysosomal proteins (CD68, CTSA, CTSB, CTSD, CTSZ, RAB7), HIF1A target proteins (ALDOA, GAPDH, LDHA, PKM) and lipid-associated proteins (LGALS3BP, LRPAP1, APOE) displayed upregulation, with similarities in protein expression to the transcriptomic DAM signature (Keren-Shaul et al., 2017).

Additionally, chronic activation of microglia can lead to prolonged inflammation from the release of pro-inflammatory cytokines and chemokines. Quantitative mass spectrometry-based proteomics performed on microglia isolated from 6 month old mice in normal (WT), acute neuroinflammatory (LPS) and neurodegenerative (5xFAD) states identified differentially expressed proteins involved in aberrant lipid trafficking, altered lipid metabolism and ATP metabolism as being enriched in 5xFAD microglia (Rangaraju et al., 2018). Interestingly, many LPS-upregulated microglial proteins were also increased in 5xFAD microglia (including CLU, NUDT2, GLIPR2, DIABLO and CSTF2), highlighting the chronic pro-inflammatory nature of microglia that may be involved in AD progression.

Much of our understanding of microglia in AD from a proteomics perspective has been gained from mouse models, however these models are often limited to studying only one element of AD pathology and as a result do not fully replicate the complexity of the disease (Yokoyama et al., 2022). Proteomics performed on cerebrospinal fluid (CSF) and homogenised brain tissue from human AD donors discovered two modules of co-expressing proteins correlating with microglia; one module enriched for gene products within AD risk loci and increased sugar metabolism in AD (M4), and another module enriched with endothelial and extracellular matrix (ECM) proteins (M5) (Johnson et al., 2020). This study highlights the extent of proteomic reprogramming of microglia in AD and the potential of dysregulated microglia metabolism contributing to disease progression. Microglia dysregulation of metabolism in AD has been shown previously via Trem2 mutations and defective mTOR signalling at the transcript level (Ulland et al., 2017), therefore making the modulation of microglia metabolism a promising therapeutic target.

#### Microglia in ageing

The major risk factor in many neurodegenerative diseases is ageing (Hou et al., 2019). Ageing profoundly affects immune cell function, with macrophages exhibiting a primed inflammatory state, loss of phagocytic capacity and lack of plasticity (Hickman et al., 2013; Olah et al., 2018; Moca et al., 2022). These phenotypic changes extend to microglia, which show age-related increases in inflammatory cytokine release even without stimulus (Conde and Streit, 2006; Moca et al., 2022). It is thought that age-related changes in microglia are a major contributor to cognitive decline and neurodegenerative diseases (Lyman et al., 2014).

It has therefore been an important endeavour to proteomically characterise ageing microglia to understand how they convey risk for neurodegenerative diseases. Microglia isolated and cultured from young (3–5 months) and old (20–24 months) mice revealed age-related increases in protein expression associated with inflammation, antigen presentation, metabolism and oxidative stress and decreases in DNA repair and transcription (Flowers et al., 2017). These changes were associated with reduced mTORC2 activity and utilisation of fatty acid oxidation from glycolysis. It is important to note that *in vitro* culture and media components have a significant impact on cellular bioenergetics that may not reflect the *in vivo* environment (Lloyd et al., 2022). Indeed, more recent comparisons of young and old mouse microglia *in vivo* have identified upregulated mTORC1 as a major driver of age-associated increases in inflammatory protein translation (Keane et al., 2021). Comparison of young (6 months), middle-aged (11 months and 15 months) and old (23 months) mice showed age-related increases protein expression of CD11b, APOE, CD45, CLEC7A, TLR2, CD11c and SPP1, all of which are also associated with the DAM phenotype (Keren-Shaul et al., 2017). mTOR inhibition by rapamycin has been shown to prolong life expectancy in lower organisms (Mannick and Lamming, 2023), and mTORC1 signalling is lower in longer lived rodents (Sharp and Bartke, 2005), highlighting mTOR as a potential therapeutic target for detrimental age-related physiological changes. Proteomics from human microglia has shown similar upregulation of inflammatory pathways, cellular stress and protein synthesis in aged compared to younger microglia (Olah et al., 2018). Interestingly, IGF1R activity was also significantly upregulated in aged human microglia (Olah et al., 2018), with IGF1 being a major activator of the mTOR pathway also found to be upregulated in ageing mouse microglia (Keane et al., 2021). Other growth factors such as NGF and BDNF also increase in the ageing brain (Katoh-Semba et al., 1998), both of which are also activators of mTOR, and may also contribute to aged-dependent microglia increases in mTOR signalling and inflammatory priming. Understanding the cellular source of these growth factor releases, reasons for their increase with age and if similar observations can be seen in neurodegenerative diseases will be important avenues of investigation.

#### Microglia in demyelination

Microglia are pivotal for remyelination and play a central role in demyelinating diseases like MS. Understanding how to harness the pro-regenerative properties of microglia to prevent demyelination and/or restore myelin represents an exciting avenue of research (Miron et al., 2013; Lloyd et al., 2019) and a potential novel therapeutic target. Brain lysates from cuprizone and experimental autoimmune encephalomyelitis (EAE), two mouse models of chronic demyelination, were processed for tandem mass tag (TMT) and label free proteomic analysis (Oveland et al., 2021). Enrichment of microglia proteins CD11b and HEXB, galectins, cathepsins and complement proteins was detected in cuprizone mice, corresponding to the observed microgliosis by histology. Although this study identified overall brain proteomic changes in response to demyelination, analysis of purified microglia populations to identify microglia-specific proteomic reprogramming will greatly enhance our understanding of molecular mechanisms underpinning microglia involvement throughout such diseases.

**Figure 2:**
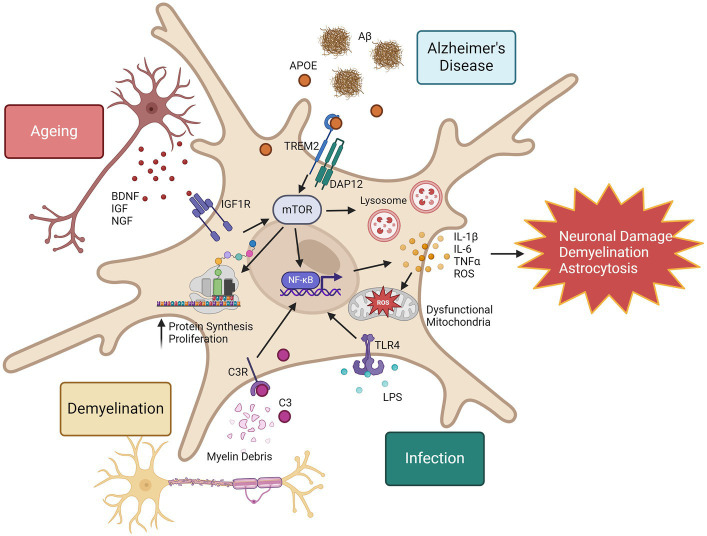
Proteomic responses to microglia in disease, infection, and ageing. Microglia in response to inflammatory stimuli can rapidly increase their expression of inflammatory proteins. One example of the mechanisms that drive this is the mTOR pathway. mTOR is also a regulator of NF-κB activity (Dan et al., 2008), leading to increased expression of a wide range of inflammatory cytokines and reactive oxygen species (ROS). Consequences to excessive ROS production include mitochondrial damage and dysfunction, and chronic release of inflammatory cytokines can promote neuronal damage, demyelination and astrocytosis. mTOR represents a promising therapeutic target to regulate microglia responses in ageing and disease. This image was generated using biorender.com.

In summary, although proteomic evaluation of microglia in disease is limited, we have insight into the proteomic reprogramming that occurs in microglia in response to disease stimuli. Microglia show both general and context-specific proteome changes, summarised in Figure 2. One commonality in microglia activation is the mTOR pathway. In ageing, increased mTOR activity is associated with chronic inflammation (Keane et al., 2021), however it has also been shown to improve amyloid beta clearance in the 5xFAD model by regulation of lysosomal activity (Shi et al., 2022), highlighting the importance of detailed pathway analysis with proteomics to provide functional context. Further proteomic evaluation of microglia in diseases may reveal novel therapeutic targets to prevent chronic microglia inflammatory responses and harness the regenerative capabilities of microglia in the CNS.

### Future of proteomics

A major aim for microglia proteomics is to understand the heterogeneity of phenotypes and disease responses at the single cell level, with functional resolution that cannot be captured with scRNAseq. Spatial biology is becoming increasingly necessary in fields of study such as neurodegeneration and oncology, as spatial techniques allow for the study of the localisation and interaction of mRNA or proteins within cells and tissue domains of interest. For the study of diseases with multiple cellular mechanisms interacting with pathologies, such as AD, spatial techniques are essential for understanding the complexities of these biological processes and disease mechanisms. Laser Capture Microdissection Spatial Proteomics (LCM-MS) allows any cell-types or tissue domains of interest to be isolated via laser capture and processed for mass spectrometry (Mund et al., 2022). As mass spectrometry sensitivity has improved over the years, smaller volumes of tissue are required per sample to effectively interrogate the proteome. Due to this, LCM-MS is a very promising technology that will be able to be increasingly utilised to isolate more specific cells/domains, even based on morphological or pathology-proximity levels.

Alongside these existing spatial proteomics techniques, more sophisticated *in-situ* proteomics methods are currently being developed and deployed, including NanoString’s new GeoMx protein panels, which allow the profiling of hundreds of protein targets simultaneously with spatial resolution. This combination of RNA and protein assays within the same tissue can allow for powerful spatial multi-omics analysis (Ignatov et al., 2023). Cellular Indexing of Transcriptomes and Epitopes by sequencing (CITE-seq) is a method that combines single-cell droplet RNA sequencing with antibody-based protein profiling (Stoeckius et al., 2017). CITE-seq currently only captures cell-surface proteins, however future developments could allow for greater multiplexing and profiling of intracellular proteins allowing for deeper cellular interrogation. Nanodroplet processing in one-pot for trace samples (NanoPOTS) also allows for sensitive proteomics on samples by downscaling volumes to less than 200 nL to minimise non-specific adsorption of proteins and peptides to surfaces, a common issue in small-sample processing. This technique can allow recovery of as many as 3,000 proteins from 10 cells (Zhu et al., 2018). This innovation, coupled with its compatibility with ultrasensitive liquid chromatography-mass spectrometry, allows nanoPOTS to achieve deep proteome profiling, potentially on a single-cell basis. Cellenion’s cellenONE platform also aims to address the challenges of working with small volume samples by isolating and dispensing individual cells before processing and running mass spectrometry (Woo et al., 2021).

## Discussion

Proteomic analysis generates important functional and translational insights into the roles of microglia in health and disease. Microglia are vital regulators of brain homeostasis with complex and variable roles in both the protection and detriment of CNS health. Proteins are the mechanistic indicators of function, therefore understanding dysregulated protein pathways present in disease microglia may highlight novel therapeutic strategies. Microglia-targeted therapies represent a promising avenue for neurodegenerative disease modulation (McKee et al., 2023) and therefore it is vital that we uncover disordered protein signalling that can be targeted. Proteomics allows us to predict disease kinetics and uncover post-translational changes that are not appreciated by transcriptomics, therefore providing greater translational relevance. As single cell transcriptomics has revolutionised our understanding of the diversity of heterogeneous responses of microglia, it is vital to gain such depth of understanding of their proteomic diversity. Advances in instrument sensitivity, sample processing (Woo et al., 2021; Derks et al., 2023) and machine learning (Churguransky, 2021; Jumper et al., 2021) mean that high resolution single cell proteomics for microglia studies is an exciting future avenue to further uncover the nuances of microglia responses in health and disease.

## Author contributions

ED: Data curation, Writing – original draft, Writing – review & editing. AL: Conceptualization, Supervision, Writing – original draft, Writing – review & editing.
